# Affective Episodic Memory System for Virtual Creatures: The First Step of Emotion-Oriented Memory

**DOI:** 10.1155/2021/7954140

**Published:** 2021-10-20

**Authors:** Luis Martin, Jonathan H. Rosales, Karina Jaime, Felix Ramos

**Affiliations:** ^1^Department of Computer Science, Center for Research and Advanced Studies of the National Polytechnic Institute, Zapopan 45019, Mexico; ^2^Faculty of Science and Technology, Autonomous University of Guadalajara, Zapopan 45129, Mexico

## Abstract

Episodic memory and emotions are considered essential functions in human cognition. Both allow us to acquire new knowledge from the environment, ranging from the objects around us to how we feel towards them. These qualities make them crucial functions for systems trying to create human-like behaviour. In the field of cognitive architectures (CAs), there are multiple studies covering memory and emotions. However, most of them treat these subjects in an isolated manner, considering emotions only as a reward signal unrelated to a retrieved experience. To address this lack of direct interaction, we propose a computational model that covers the common processes that are related to memory and emotions. Specifically, this proposal focuses on affective evaluations of episodic memories. Neurosciences and psychology are the bases of this model. That is, the model's components and the processes that they carry out on the information they receive are designed based on evidence from these cognitive sciences. The proposed model is a part of Cuáyóllótl, a cognitive architecture for cybernetic entities such as virtual creatures and robots. Case studies validate our proposal. They show the relevance of the integration of emotions and memory in a virtual creature. The virtual creature endowed with our emotional episodic model improves its learning and modifies its behaviour according to planning and decision-making processes.

## 1. Introduction

Knowledge is the most powerful tool that human beings have for dealing with everyday life. Experience provides much of our knowledge in multiple situations. This experience prepares us for similar situations in the future, when we can improve our responses to obtain better results.

In human beings, emotions play a unique role because they influence the storage and retrieval of knowledge. Specifically, emotion-oriented memory is a specialized process for storing and retrieving emotional evaluations, which allows emotions to be generated or consolidated from the combination of previous knowledge and current perceptions of the environment. Two specific cognitive processes are involved in this process: emotions and memory.

Both the cognitive process of emotions and the memory process are regularly studied in the literature independently, due to the complexity of understanding them and the neural distribution they have within the brain. We have relied on neuroscientific and psychological evidence to identify and define these cognitive processes in this research. Based on this information collected from the literature, in this proposal, we present the identified brain structures involved in both cognitive processes.

We look for a way to store affective evaluations because this is considered the first step of emotional processing [1]. We propose software cognitive memory architecture oriented toward the affective process inspired by the behaviour of the human brain structures involved. We call this proposed model affective episodic memory. That is, this proposal is a system that allows learning and memorizing including affective evaluation, considering the possibility of including inputs and outputs to the different cognitive functions that make up the human mind. In other words, our proposal can be included in a cognitive architecture biologically inspired.

A virtual creature provided with the proposed cognitive architecture was placed in a controlled environment to verify the proposal's results. Endowing virtual creatures with mechanisms such as affective episodic memory allows natural behaviours to be observed in these creatures. We observed biases in decision-making in response to certain perceived stimuli in the environment that were endowed with affective values from the retrieval of information from memory generated in previous highly affective experiences. This behaviour is similar to human behaviour.

We tested the proposal through three case studies. In these case studies, we placed the virtual creature in a controlled virtual environment, including multiple emotional stimuli, which generated the agent's emotional responses. In the first case, the agent navigated the world and learned the objects' affective associations in the environment. The main goal was to show whether it could retrieve the affective values correctly. In the second case, the agent learned the environment and used the affective associations to reach a goal. Here, we aimed to demonstrate how the affect could be useful to generate observable biases in the virtual creature's decisions within the virtual environment. Finally, the third case presented a set of images with different affective intensities that the virtual creature had to learn. The objective of these case studies was to evaluate how the affect can improve memory, so that the images are not forgotten.

The document is structured as follows. We start with a brief introduction to the cognitive systems involved. Then, we analyse some cognitive architectures identified in the literature that consider memory and emotion processes. Next, we describe the brain structures identified in the affective memory process. We then describe the proposed architecture based on the neural information collected. Finally, we report the implementation of the architecture and show its functionality with some case studies, culminating with a brief discussion and some conclusions.

We start by describing a little more about the cognitive processes of memory and emotions.

### 1.1. Memory

As a cerebral function, memory can be considered one of the most important cognitive functions in human beings due to its capacity to keep lasting representations acquired from the environment through a learning process. These representations reflect our thoughts, experiences, and adaptive behaviours. However, this acquisition of representations depends on three main functions: encoding, storing, and retrieval [2, 3]. The encoding creates patterns using data acquired from the environment. The data encoded in a pattern can later be stored and retrieved when required by other cognitive functions.

Regardless of the processes that it carries out, memory is not considered a unitary faculty of the mind. On the contrary, it is composed of multiple systems with different operating principles and different neuroanatomy [4, 5]. Here, we focus on the division based on the level of consciousness: declarative memory (conscious), and nondeclarative memory (unconscious), specifically on episodic memory. This memory is a type of declarative memory responsible for supporting the storage and recollection of experienced events [6, 7].

### 1.2. Emotions

Among the cognitive processes that most intrigue the scientific community is undoubtedly the process of emotions. The generation of emotions is typical of living beings since, through emotional behaviours, we can express our internal states and even engage in interspecies communication.

This process consists of three primary or essential components for its generation [1]:

1. An affective evaluation, aimed at determining the emotional value (positive or negative) of the stimuli perceived in the environment
2. An internal emotional state, which is generated by affective evaluations and biases in our responses in the environment
3. An emotional response behaviour, which expresses the internal emotional state through a deterministic behaviour

These components interact among themselves, in addition to interacting with other cognitive processes such as memory. Specifically, the link between emotions and memory has not been investigated extensively, i.e., we found few studies related to the interaction between these two fundamental cognitive processes in living beings.

Regarding emotions, we focus within this study specifically on the brain structures involved in affective evaluations since this process is considered the first stage of the cognitive process of emotions.

Our objective is to look for a way to store the perceived stimuli and the associated affective evaluations, using the memory mechanisms identified in the neuroscientific evidence: encoding, storage, retrieval, and forgetting.

## 2. Cognitive Architectures

Currently, several research groups are trying to endow virtual agents with human-like capabilities through a particular piece of software called cognitive architecture (CA). However, few projects have considered the integration of episodic memories and emotions. In this section, we present the most relevant cognitive architectures, considering episodic memory and emotions. Thus, projects such as Soar [8, 9], ACT-R [10], iCub [11], and LIDA [12] were included.

Soar is a cognitive architecture designed for the development of intelligent agents [8, 9]. In the case of memory, this project covers multiple types such as procedural memory, semantic memory, episodic memory, and working memory. In addition, Soar includes three kinds of emotional components: emotion, mood, and feeling. However, Soar has limited its exploration of emotions as they relate to other functions. It only uses feeling as an internal reward value to drive reinforcement learning. Regarding its interaction with memory, feeling does not have direct interaction with other memory modules except for working memory [9].

In general, the emotional system of Soar has not been implemented and is only part of its general model [9]. Furthermore, the fact that it is only related to working memory limits the capacity of the architecture to influence other cognitive functions such as planning, decision-making, and long-term memory.

Adaptive control of thought-rational (ACT-R) is a cognitive architecture and a theory about how human cognition works [10, 13, 14]. It contains two types of modules: the perceptual-motor and memory modules. Specifically, the memory module is divided into declarative memory for facts and procedural memory for rules. Even though the original ACT-R does not include an affective or emotional module, Dancy [15] extends it by adding the affect module. This extension provides a functional layer between the physiological and cognitive systems that is based on existing neuroscientific and psychological evidence; it also allows one to simulate some effects of homeostasis on cognition [15]. However, it only focuses on seeking behaviour, leaving memory interaction aside. And, like the original ACT-R, it does not make a distinction between episodic and semantic memory. Thus, this cognitive architecture is not able to integrate episodic memory with an emotional value.

The learning intelligent distribution agent (LIDA) model is a conceptual and computational model attempting to cover a large portion of human cognition [12, 16]. It is based on Baars's Global Workspace Theory (GWT), a theory of the role of consciousness in cognition [12, 17], and other psychological and neuropsychological theories. It consists of several types of memories, including episodic and semantic memory. In LIDA, emotions are used as drivers that motivate action selection and as modulators that affect the learning rate. Feelings are represented as nodes in perceptual associative memory and occur and play a central role in the determination of activation values throughout the model [18].

However, although LIDA models include interaction between the emotions and some aspects of the current situation, emotion does not interact directly with declarative or episodic memory. Furthermore, it is a model that has not yet been implemented [18].

iCub is an open-system 53-degree-of-freedom humanoid robot and an open-system research platform designed for the embodied cognitive system community [19]. iCub is grounded in psychology, neurophysiology, and neuroscience. It is endowed with episodic and procedural memories that allow for internal simulation to provide capabilities for prediction and reconstruction. The episodic memory component is a simple memory of visual autobiographical events. It is a form of one-shot learning and, in its present guise, does not generalize multiple instances of an observed event [20]. The affective state module receives inputs from the episodic memory and affects the iCub's motivations. These motivations (curiosity, experimentation, and social engagement), together with the action selection component, provide a homeostatic process that regulates the iCub's behaviour [19, 21].

Although it has the affective state module, it is not used to bind objects and episodes with an affective value but to decide a kind of behaviour.

In addition to the architectures presented above, there are additional projects such as DUAL-PECCS [22, 23], CLARION [24], EPIC [25], and CHREST [26, 27]. However, these architectures are more focused on cognitive abilities and knowledge representation. Therefore, they do not consider the integration of emotions with episodic memory. Also, there is a project called LEABRA [28, 29] that considers the integration of emotions but using a connections approach.

Overall, there are plenty of systems that try to embed the processes of memory and emotions in virtual creatures. However, most of these systems use predefined emotional responses, which remain invariant over time. These characteristics make virtual creatures' behaviour predicvd and unrealistic. Furthermore, they are still in the modelling stages and do not consider a direct integration with memory systems.

## 3. Neuroscientific Evidence

**Figure 1:**
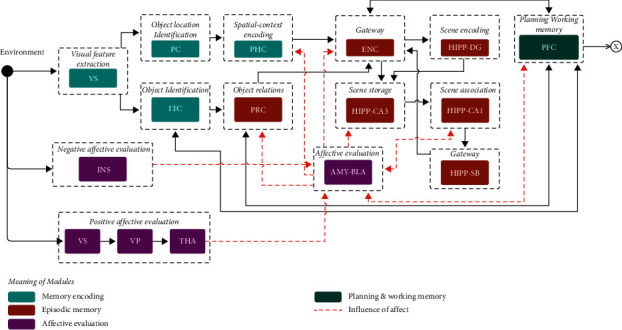
Structures involved in affective and declarative memory processes.

Memory and emotion processes are cognitive functions regularly investigated in isolation in neuroscience. There is plenty of evidence of these two cognitive functions. From this evidence, we define the set of brain structures involved in these functions and their processing.  Figure 1 presents the common areas between the cognitive processes of memory and emotions and the brain areas involved in each of these processes.

Next, we present the neuroscientific evidence about the brain structures involved in affective episodic memory to understand the processes associated with these areas.

### 3.1. Visual Areas for Object Identification (VS)

This represents a set of different brain structures related to the object identification process. These areas generate one of the main inputs of sensory information to the cerebral cortex. The eye captures visual data from the environment. The lateral geniculate nucleus relays and filters the data through different paths in the brain. The primary visual cortex makes segmentation of objects in the scene. The secondary visual cortex encodes objects. Then, the information is conveyed to the inferior temporal cortex and premotor cortex [30–35].

### 3.2. Inferior Temporal Cortex (ITC)

This area is responsible for conveying encoded objects to the memory system; it selects and reports the classes that identify the object in the current scene [31, 32, 34, 35]. It plays a role in the recognition and categorization of visual objects. Also, it helps to form high-level object representations through the synthesis of features. The groups of neurons in the ITC encode faces and categories of objects [36]. The ITC can be subdivided into the anterior, medial, and lateral areas. The anterior part helps in retrieving functional information about objects [37]. The lateral part stores information about living objects [33]. The medial part is related to inanimate objects [33].

### 3.3. Posterior Parietal Cortex (PC)

The posterior parietal cortex is part of the dorsal stream and processes objects' spatial properties (position, size, and movements). Therefore, all elements are required to perform spatially guided gestures. The inferior parietal lobe (IPL) covers the ventral aspect of the PPC. It has a role in visuospatial working memory by maintaining maps of the entire visual field with essential information [38]. Its neurons can provide the spatial information required for directing attention to a salient stimulus in a complex scene [39] and retaining a memory trace of the position of essential elements perceived in the visual scene by maintaining and updating their representation on oculocentric maps renewed after each new eye movement [38]. Together, the dorsolateral prefrontal cortex (DLPFC) and PPC are involved in keeping spatial information in memory over a short time [40].

### 3.4. Parahippocampal Cortex (PHC)

This performs visuospatial processing related to scene perception, spatial representation (egocentric and allocentric), and navigation [41, 42]. It is also involved in contextual association processes such as binding a target item to the surrounding context and supporting recollection by encoding and retrieving contextual information [41, 43, 44].

### 3.5. Perirhinal Cortex (PRC)

The perirhinal cortex is an association area; it receives unimodal and polymodal sensory inputs [45]. It works as an interface between the neocortex and the hippocampus through the connections with the entorhinal cortex [45]. It is related to object recognition and memory for items: encoding, storing, and retrieving [43, 46]. Because it has high connectivity with the amygdala [47–49], it could be a place for item-emotion associations [47, 49, 50]. The perirhinal cortex has a mostly integrative role. It is a semantic hub and has object-specific information. It encodes and retrieves abstract object-specific information [33, 34, 37, 51–54].

### 3.6. Entorhinal Cortex (ENC)

This is a part of the medial temporal lobe, an important area for declarative memory [55, 56]. It is the major gateway to the perirhinal cortex and the hippocampus [45, 48, 52, 56]. Like the perirhinal cortex, it receives massive projections from the basolateral amygdala [48]. The amygdala and the entorhinal cortex show positive correlations during the encoding of emotionally arousing images, but not neutral ones [57–59].

### 3.7. Hippocampus (HIPP)

The hippocampus is the most important area for declarative memory. It helps in the object-recognition process and the encoding of new memories [52, 54, 60]. It is involved in the acquisition of both semantic [37] and episodic memories [6, 61–64]. It is also critical for the retrieval of episodic memories, but not semantic memories. This structure is involved in the processing of spatial or contextual information [63, 65, 66] and the creation of item-in-context associations to form episodic memories [49, 50]. It is associated with emotional memory since damage to this structure can cause memory loss, lack of expressiveness, and even inability to generate emotions [67, 68]. It receives information from the perirhinal cortex through the entorhinal cortex [49, 51, 64]. The connections with the amygdala [69] highly influence memory processes such as encoding and retrieval. It consists of the subareas dentate gyrus, subiculum, and cornu ammonis (subdivided into CA1, CA2, CA3, and CA4).

#### 3.7.1. Cornu Ammonis 3 (CA3)

This area is involved in the integration of multimodal information coming from the entorhinal cortex [65]. It integrates spatial and nonspatial information in object-place or event-context representation [70, 71]. Pattern completion grounds the retrieval in CA3 [72, 73]. This process takes partial input and transforms it into the entire stored event. Thus, it plays a role in encoding and retrieving episodic memories [65, 71, 72]. It performs pattern separation with the dentate gyrus to provide a potential neuronal substrate for disambiguation of overlapping memories in the hippocampus [73].

#### 3.7.2. Cornu Ammonis 1 (CA1)

Together, CA1 and CA3 play an important and complementary role in memory processes for episodic memory [72]. CA1 keeps the representation of the episodes; it also helps in encoding and retrieving the global context [74]. Thus, it is a part of the emotional memory process, particularly in the association of affective impulses with certain stimuli [75]. It performs pattern separation to differentiate between scenes and scene completion [72, 73, 76].

#### 3.7.3. Dentate Gyrus (DG)

This structure contributes to pattern separation work, a process required to differentiate between similar memories [77]. The DG generates new patterns that represent new memories; these patterns help in the retrieving process. The unknown stimuli that lead to the generation of the patterns come from the entorhinal cortex, and DG output is the principal input of the hippocampus [64].

#### 3.7.4. Subiculum (SB)

This area is involved in the generation of new episodic memories and represents the main output from the hippocampal formation [64]. There is evidence showing that it participates in the processes of recovery of emotions and emotional evaluations [78].

### 3.8. Amygdala (AMY)

The amygdala helps in all the aspects of encoding and retrieving emotional items. Emotionally aversive scenes specifically enhance recollection rather than familiarity [79]. The amygdala has a role in the consolidation of long-term memories due to its connections with the hypothalamus-pituitary-adrenal axis (HPA) by influencing the release of stress-related hormones and neurotransmitters [57, 59, 80–82]. It is massively connected to the perirhinal cortex, entorhinal cortex, and hippocampus [49, 69, 75, 83]. These connections may enhance the memory processes for emotionally salient events [75, 79, 81, 83, 84]. These memory enhancements change how a scene is perceived by making it more vivid and providing a positive or negative emotional stamp [65, 69, 75, 85]. The amygdala generates the emotional effects on episodic memory and is more active during the encoding and retrieval of emotional memories [49]. There is evidence that collaboration between this structure and other limbic structures enables the affective assessment of the environment [75, 86]. It is involved in both the generation of affective responses and the regulation of affective states [87–89]. It modulates an item's emotional properties, supporting the binding of items from the perirhinal cortex to emotional information [49, 50].

#### 3.8.1. Basolateral Amygdala (BLA)

The BLA enhances the memory of emotionally arousing experiences [79] through the regulation of neural plasticity and information storage processes in other brain regions [90]. Its activity enhances the interaction between the perirhinal and entorhinal cortices [91], i.e., it facilitates the association and storage of high-arousal signals in the rhinal cortex (PRC and ENC). This modulation is probably related to the processing and storage of emotional memories [48, 91]. Also, the activity level in this area can modulate the strength and intensity of emotional memories through the enhancement of the CA1 neurons' excitability [92]. This structure also modulates memory consolidation in the hippocampus [83, 93] and is related to the enhancement of memory processes for emotionally salient events (episodic memory) [83, 90].

Moreover, this structure is very much involved in Pavlovian or classical conditioning [94, 95], affective conditioning [96], the associative reward process [96–98], and fear conditioning [99, 100]. Maren [99], however, shows that classical conditioning exists even without this structure although it is more difficult to generate.

Anatomically, it is considered the nociceptive nucleus of the AMY and is involved in pain processing. However, it seems to be activated only in the combination of pain and the current affective evaluation (guided by emotional states) [101].

### 3.9. Prefrontal Cortex (PFC)

This area is related to several executive control functions. It is involved in working memory processes, control of semantic memory, episodic memory, and selective attention [2, 33, 37, 102]. It consists of multiple subregions, such as the DLPFC, ventrolateral prefrontal cortex (VLPFC), medial prefrontal cortex (MPFC), and orbitofrontal cortex (OFC), related to specific cognitive control processes [103, 104]. Neuroimaging establishes that these regions are involved in retrieving knowledge, maintaining behavioural goals, task-switching, and adaptively manipulating information held in short-term memory [105–108]. It also acts as the interface with long-term memory [109]. The PFC retrieves and inhibits memory information within a given context. Some authors have identified that the PFC may encode the stimuli received in an abstract representation that are useful to guide behaviour [110, 111]. This means that the PFC works with high-level memory representations such as goals, task rules, or categories [112–114]. Similarly, it is involved in the processes of cognitive reward [115], in affect (positive and negative) [116] and in the generation and control of emotions [117–119].

### 3.10. Insula (INS)

This brain structure has a strong involvement in pain processing [100, 116, 117, 119–121] since it is a part of the pain matrix, which is a system made up of several brain structures aimed at generating cognitive or internal pain [122]. It is also a part of the nociceptive system responsible for the perception and identification of pain [100, 116, 118, 120, 123, 124].

### 3.11. Ventral Striatum (VS)

This structure is involved in motor responses directly related to stimuli perceived with rewards [97, 98]. It is a part of the brain's dopaminergic system [125]. The processing of the reward in terms of both sending signals and the output activates this structure [126].

Anatomically, within this structure is the nucleus accumbens, involved in the mediation of the motivational effects of relevant emotional stimuli [68], in late or expected reward (“wanting”) [94, 115, 127–130], in the modulation of unconditioned behaviours such as hunger and locomotion and learned behaviours [94, 127], in procedural learning oriented to reward [68, 96, 131] and in the placebo effect [127, 128].

### 3.12. Ventral Pallidum (VP)

This structure is involved in reward processing and in emotional processing [130, 132–135]. It is a part of the dopaminergic system of the brain [125, 134].

Some studies place this structure, together with the nucleus accumbens, in hedonic processing, which when stimulated generates an increase in the “liking” and “wanting” of the reward process in various physiological needs, such as hunger and thirst [130, 132–135]. Also, a strong involvement of this structure is observed in the inhibition of the ventral tegmental area (VTA) [96].

### 3.13. Thalamus (THA)

This brain structure is activated in affect-oriented studies [30, 88, 136], both in painful stimuli [117, 121] and in pleasure stimuli [94, 96, 133, 137].

The main hypotheses place this structure as a relay responsible for distributing incoming stimuli to the various upper areas of the brain. Some evidence shows that it participates in the filtering of stimuli, determining their relevance, based on the intensities of inputs received from the perceptual regions of the brain [86, 118]. In reward studies, its operation is oriented toward retransmitting information between structures, forming information loops [94, 95, 133, 137]. In addition, atrophies in this structure have been shown to increase chronic pain [118].

## 4. Bioinspired Model of Emotional Episodic Memory

In this section, we present a computational-oriented bioinspired model built from the functions performed by each brain structure identified previously. We then make a detailed description of its components and their processing. We conclude by showing the types of information flow inside the model. Overall, the proposed model is developed following the methodology for cognitive architecture construction proposed by Jiménez et al. [138].

### 4.1. Computational Model and Components

**Figure 2:**
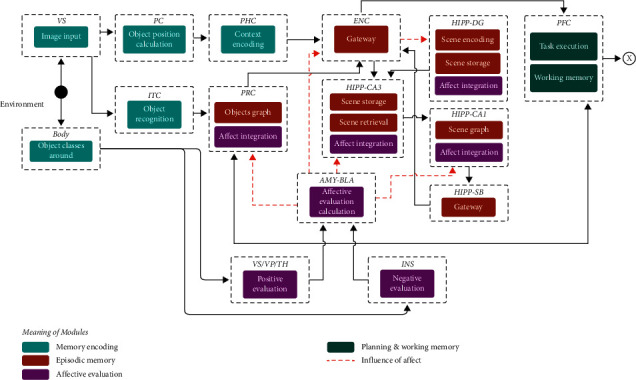
Affective episodic memory model.

The proposed system is built from a subset of the brain structures and connections identified from the neuroscientific evidence of Section 4 (see Figure 1). The components (structures) that make up the proposal are those directly associated with affect and episodic memory. Each one is associated with its biological counterpart, preserving its operations and relations with other areas. When we say preserving its operations, we mean that each component performs multiple computational operations according to what they do in nature. Likewise, the interaction among them is preserved from the literature (see Figure 1).  Figure 2 shows the resulting proposal. The following is a detailed description of each component.

#### 4.1.1. Environment

The environment represents everything that surrounds the virtual agent. It can be the real world captured from a camera or a 3D world created to immerse the agent. It sends the body a set of IDs representing the objects that surround the agent.

#### 4.1.2. Body

This receives and transfers to the affective evaluation components the class ID of all the objects that surround the virtual agent.

#### 4.1.3. Visual System (VS)

This component starts the encoding process. It grabs an image from the environment and sends it to the modules linked to visuospatial processing. This operation is too general and does not reflect the processes of the visual cortex in full detail. We do this for simplicity because those processes are beyond the scope of this article. However, Gonzalez-Casillas et al. [139] describe these processes in our cognitive architecture.

#### 4.1.4. Parietal Cortex (PC)

This is part of the dorsal visual processing. It performs operations related to the extraction of the spatial properties of the stimuli. The PC carries out segmentation processing to identify the objects in the image and the centre points of each one. This module assigns a temporary ID number to the objects for future processing.

#### 4.1.5. Inferior Temporal Cortex (ITC)

This is part of the ventral visual processing. However, unlike the PC, it is not focused on spatial properties but on recognition. This module performs segmentation to extract objects and to execute a recognition process for each extracted object. Like the PC, it assigns a temporary ID to the objects. In general, this area knows what we are watching, while the PC knows where it is located.

#### 4.1.6. Parahippocampal Cortex (PHC)

This module is also involved in spatial encoding, specifically in creating maps that represent the spatial context. It transforms the set of objects and locations received from the PC into an occupancy grid. The grid is used to create a symbolic pattern that keeps the spatial relation (context) among the objects. Chang and Jungert [140] proposed this representation pattern.

#### 4.1.7. Perirhinal Cortex (PRC)

This is related to multiple associative processes. First, it creates binary relations between the recognized objects in the scene. The PRC has connections with the affective module (AMY-BLA), which allows it to assign a positive and negative value to each object. Moreover, the intensity of these values directly affects the weight of the relation among the objects. We use a graph to store both types of relations. The information contained here is useful in future cognitive processes.

#### 4.1.8. Entorhinal Cortex (ENC)

This component works as a bridge, i.e., it retransmits the data received to their next destination.

#### 4.1.9. Dentate Gyrus Hippocampus (HIPP-DG)

This assembles the final pattern that represents the memory of a scene. It combines the data coming from the PHC with the object-recognized classes from the ITC. These data form a 2DString pattern that keeps the spatial context but does not contain data about the type of objects. Also, the DG assigns the affective values coming from the BLA to the created scene, and like in the PRC, the intensity of this value affects the strength of the memory. Each created memory gets a unique ID and is stored.

#### 4.1.10. Cornu Ammonis 3 Hippocampus (HIPP-CA3)

This stores the memory created in the DG, acting as a backup of knowledge. Like the DG, it performs the same integration of data coming from the PHC, ITC, and BLA, but the pattern is used only to search for data in the backup. This means it searches in parallel while the DG is storing. Due to its connections to the BLA, this module can improve the search for emotional memories by grouping the scenes that have an affective value in a specific range. Then, those memories that belong to the group of the current BLA state are prioritized in the search.

#### 4.1.11. Cornu Ammonis 1 Hippocampus (HIPP-CA1)

This component is responsible for the creation of the internal representation of the environment. It links each scene pattern coming from the DG to create multiple sequences of scenes; these sequences represent episodes. Thus, it stores a graph representation of the world. Like in the PRC, every pair of associated memories is affected by the intensity of the affective value coming from the AMY.

#### 4.1.12. Subiculum Hippocampus (HIPP-SB)

Like the ENC, this area works as a data bridge. It retransmits all the data received to the desired destination.

#### 4.1.13. Prefrontal Cortex (PFC)

As we state in the biological evidence, the PFC is related to several cognitive processes such as working memory and cognitive control. Thus, this module includes all the planning and decision-making logic and temporarily stores the knowledge retrieved from the PRC, CA3, and CA1.

#### 4.1.14. Insula (INS)

This is responsible for providing negative affective evaluations, aimed at generating pain from estimates perceived in the environment.

#### 4.1.15. Ventral Striatum and Ventral Pallidum (VS/VP)

This is responsible for determining the reward given to a perceived stimulus from the affective and motivational evaluations, in addition to current physiological needs that would increase or decrease the reward of a perceived stimulus.

#### 4.1.16. Basolateral Amygdala (AMY-BLA)

This nucleus of the AMY is responsible for generating an affective evaluation associated with the stimuli perceived in the environment through the emotional values recovered and current affective perceptions.

### 4.2. Processing Stages and Interaction Flows

Now that we have defined the general functions carried out by each component of our model, we proceed to show their interaction and how they process the exchanged data. The multiple pathways of communication we propose enable the execution of specific emotional memory stages: encoding, storage, and retrieval. Although we present these pathways individually, it is worth mentioning that, to reach the whole memory functioning, these pathways must all run simultaneously in a distributed and concurrent manner; moreover, we need to be aware that they are also dependent on each other.

#### 4.2.1. Encoding

**Figure 3:**
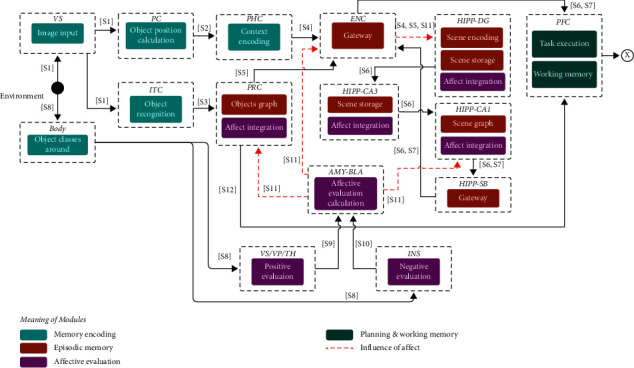
Flow diagram of the components associated with the retrieval of affective memories. The labels on the lines show the type of spike that is sent between them (see Table 1).

This stage involves the interaction between the components related to the transformation of the data from the environment into a format that can be stored by the other components. In Figure 3, we show that the modules VS, ITC, PC, and PHC extract the visuospatial properties of the image acquired from the environment. At the same time, body, INS, VS/VP, and BLA calculate the affective values using the sensed environment. Finally, PRC, ENC, DG, CA3, CA1, and SB integrate these data into a single representation that can be stored and subsequently retrieved. On the other hand, PFC stores the pattern created recently.

#### 4.2.2. Retrieval

**Figure 4:**
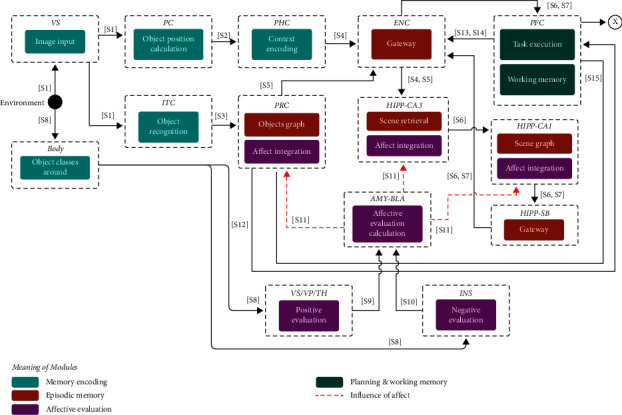
Flow diagram of the components associated with the retrieval of affective memories. The labels on the lines show the type of spike that is sent between them (see Table 1).

This stage comprises two types of retrieval. The first one is triggered by the data coming from the environment. Like encoding, retrieval implies the interaction of the components VS, ITC, PC, and PHC to extract the visuospatial features. Then, PRC, ENC, CA3, CA1, and SB integrate the data into a pattern that is used only for searching, not for storing. In this case, body, INS, VS/VP, and BLA are used by the storage modules to refine the searching of memories with a similar level of affective values, and PFC stores the retrieved knowledge. Also, the storage components update the stored affective values with the values perceived. Finally, the second type depends on a query generated by PFC, which triggers the retrieval into ENC, PRC, CA3, CA1, SB, and returns to PFC.  Figure 4 shows the participating components.

### 4.3. Process Formalization

As we described earlier, each component is responsible for performing a specific task in the system. In this section, the functions performed by each module are formalized. However, it is worth clarifying some concepts beforehand. We will consider a scene as the current event (image) that the agent sees an object as one of the elements that compose the scene and a class as an object category (e.g., chair, dog, pizza, etc.).

#### 4.3.1. Image Input

This grabs an image from the virtual environment, and the image is used as the main input into the system. We denote the input image *I* as

(1) $$\begin{array}{c} I=\left[\text{byte array of a JPEG encoded image}\right]. \end{array}$$

#### 4.3.2. Object Position Calculation

This receives the image and identifies the location of the existing objects. First, the Yolo [141] object detection algorithm detects the objects and their origin points. Then, the centre points (*x*, *y*) of each object are calculated using the origin, and a detection ID is assigned to it. Finally, it sends the list of centre points (*x*, *y*) and ID as output.

Let *p* be the number of detected objects, then *C* is a set of tuples that relates an object to its centre location.

(2) $$\begin{array}{c} C=\backslash \left\{\left(1,x_{1},y_{1}\right),\ldots ,\left(p,x_{p},y_{p}\right)\backslash \right\}, \end{array}$$

where *x*, *y* ∈ *ℝ*,  *p* ∈ *ℕ*.

#### 4.3.3. Spatial Context Encoding

This simplifies the scene representation using an occupancy grid *G* created from the set of tuples *C*.

Then, let *P* be the set of IDs assigned to the points:

(3) $$\begin{array}{c} P=\backslash \left\{1,\ldots ,p|p\in \mathbb{N}\backslash \right\}, \end{array}$$

and *G* be an *m* × *n* matrix:

(4) $$\begin{array}{c} G=\left[\begin{array}{ccccc} z_{11} & z_{12} & z_{13} & \cdots & z_{1n}\\ z_{21} & z_{22} & z_{23} & \cdots & z_{2n}\\ \vdots & \vdots & \vdots & \ddots & \vdots \\ z_{m1} & z_{m2} & z_{m3} & \cdots & z_{mn} \end{array}\right], \end{array}$$

where *z*_{*ij*}=0(empty) or *z*_{*ij*} ∈ *P* (occupied).

Finally, this matrix is converted into a symbolic representation of the scene using the pattern proposed by Chang and Jungert [140] and the method of episodic memory encoding proposed in Martin [142]. As we mentioned earlier, this pattern considers the spatial location but not the classes of objects. Thus, we call this a scene over *P*.

(5) $$\begin{array}{c} {\text{Scene}}_{P}=2{\text{DString}}_{P}. \end{array}$$

#### 4.3.4. Object Recognition

This receives the image  *I* and performs a recognition process to extract the objects in the scene and their respective classes. Similar to the object position calculation, this process is performed by the Yolo algorithm, but in this case, considering the classes of the detected objects instead of the locations.

The output is a set of object IDs, given by the detection order and their associated class.

Again, let *p* be the number of detected objects and *T* be the set of possible belonging classes taken from a dataset. Then, let *O* be the set of tuples that relate an object to a class.

(6) $$\begin{array}{c} O=\backslash \left\{\left(1,t_{\alpha }\right),\ldots ,\left(p,t_{\iota }\right)\backslash \right\}, \end{array}$$

where *t*_*n*_ ∈ and‖*O*‖=*p*.

#### 4.3.5. Negative Evaluation

This is responsible for assigning the affective values of pain to the scene objects. This value is independent for each stimulus identified in the scene and is defined as an affective parameter excitement of pain es_pain_; its value is among the parameters found between 0.0 < es_pain_ < 1.0. Given the set of stimuli perceived in the scene, we can build a set of excitations of affective parameters of pain ES_pain_, where ∀es_pain_ ∈ ES_pain_.

#### 4.3.6. Positive Evaluation

This is responsible for assigning the affective values of pleasure to the scene objects. This value is independent for each stimulus identified in the scene and is defined as an affective parameter excitement of pleasure es_pleasure_; its value is among the parameters found between 0.0 < es_pleasure_ < 1.0. Given the set of stimuli perceived in the scene, we can build a set of excitations of affective parameters of pleasure ES_pleasure_, where ∀es_pleasure_ ∈ ES_pleasure_.

#### 4.3.7. Affective Evaluation Calculation

This is responsible for providing an affective assessment from the environment. Given the set of excitations of the affective parameters of pleasure ES_pleasure_ and pain ES_pain_, we can construct the general set of affective excitations ES_*ρ*_, where *ρ*={pleasure, pain} and ES_pain_ ∪ ES_pleasure_=ES_*ρ*_. To continue to represent the functionality of a brain structure, for all excitatory inputs, there is a relevance within the structure, so an affective value as_*ρ*_ will be given by the sum of the affective parameter excitement ES_*ρ*_ multiplied by the set of relevance RS_*ρ*_, so the affective value as_*ρ*_ in a certain period *t* will be given by

(7) $$\begin{array}{c} {\text{as}}_{\rho }\left(t\right)=\frac{1}{n_{\rho }}\sum_{k=1}^{n_{\rho }}{\text{rs}}_{\rho k}*{\text{es}}_{\rho k}, \end{array}$$

where *n*_*ρ*_ is the total number of elements of ES_*ρ*_, rs_*ρ*_ ∈ RS_*ρ*_, es_*ρ*_ ∈ ES_*ρ*_, and 0.0 < rs_*ρ*_, es_*ρ*_ < 1.0. Finally, we will define the set of affective evaluations parameters Pa given by all affective evaluations as_*ρ*_ such that ∀as_*ρ*_ ∈ Pa.

#### 4.3.8. Scene Encoding

This creates the final pattern that represents a scene by combining a scene over *P*(Scene_*P*_) and the set of recognized objects *O*. It replaces the temporary ID *p* ∈ *P* with the object class *t* ∈ *T* associated with *p*. Then, a scene is defined as

(8) $$\begin{array}{c} \text{Scene}=2{\text{DString}}_{T}. \end{array}$$

Taking the affective values as_*ρ*_ coming from BLA, we can define an affective scene as the association of a scene pattern with these affective values:

(9) $$\begin{array}{c} {\text{Scene}}_{a}=\left({2\text{DString}}_{T},{\text{as}}_{\rho }\right). \end{array}$$

#### 4.3.9. Object-Affect Association

This uses the received set *O* of object classes to create a graph of relations among the objects. The sensory affective values mentioned earlier, as_*ρ*_, are also linked, and the connection among them is weighed. Let *O*′ be the set of object classes without repetition built from *O* and *O*^″^ be the set of classes associated with affective values. Thus, an affective object is the association between an object class with the affective values. Then, we define the affective objects as

(10) $$\begin{array}{c} O^{\prime \prime }=\backslash \left\{\left(o_{1}^{\prime },{\text{as}}_{\rho_{1}}\right),\ldots ,\left(o_{n}^{\prime },{\text{as}}_{\rho_{n}}\right)|o_{x}^{\prime }\in O^{\prime }\backslash \right\}. \end{array}$$

Thus, we define the weighted graph *G*_*o*_ as *G*_*o*_=(*V*_*o*_, *E*_*o*_), where *V*_*o*_=*O*^″^ and *E*_*o*_=*V*_*o*_ × *V*_*o*_.

Also, the relation between these objects is weighted given a function *a*_*f*_ that we will explain as follows. Then, the activation function *a*_*f*_ assigns a weight to each edge *e*_*o*_ ∈ *E*_*o*_.

(11) $$\begin{array}{c} a_{f}:E_{o}⟶\mathbb{R}. \end{array}$$

#### 4.3.10. Scene Association

Like object association, this creates relations but at the scene level. These relationships create a graph of the environment.

Let *S* be a set of emotional scenes. Then, the environment graph *G*_*s*_ is defined as

(12) $$\begin{array}{c} G_{s}=\left(V_{s},E_{s}\right), \end{array}$$

where *V*_*s*_=*S* and *E*_*s*_=*V*_*s*_ × *V*_*s*_.

Like object relations, the function *a*_*f*_ assigns a weight to the relations between scenes:

(13) $$\begin{array}{c} a_{f}:E_{s}⟶\mathbb{R}. \end{array}$$

#### 4.3.11. Storage Components

The components involved in memory storage, such as PRC, DG, CA3, and CA1, preserve the previously described data types for future retrieval of object relations, similar scenes, and scene relations. By default, they have a base level emotional value that influences the reinforcement of memory traces. This level can be modified during a time interval by the intensity of the affective values. Once the time has passed, the emotional value returns to the base level. The activation function is defined as follows.

A weight *w*_1_ that controls how much the affect is going to influence the memory trace. This value is chosen from the maximum value of the affective parameters as_*ρ*_ ∈ Pa, where *ρ*={pain, pleasure}.

(14) $$\begin{array}{c} w_{1}\left(t\right)=\max\left({\text{as}}_{\rho }\right). \end{array}$$

Weight *w*_2_ controls how much the memory activation grows based on the number of times it has been experienced. The value is calculated from the current number of repetitions rep multiplied by a scale number sc that controls the speed of growth.

(15) $$\begin{array}{c} w_{\left\{2\right\}\left(t\right)}=\text{rep}*\text{sc}, \end{array}$$

where rep ∈ *ℕ* and 0.0 < sc < 1.0.

The general weight *w*(*t*) is the addition of the affective *w*_1_ and memory *w*_2_ weights multiplied by two parameters *α*, *β* that control which value is more relevant. It means that values of alpha greater than zero lead to an improvement of memory retention (*w*(*t*)), and when *α* is zero, there is not such improvement. This parameter seeks to simulate the fact that affective values produce a memory improvement in humans. On the other hand, *β* controls how fast the repetition can improve memory (default human behaviour).

Together, these values can be used to regulate how the affect and the experience can help memory traces to be remembered. High values of *w*_1_ and *w*_2_ lead to a higher activation, thus improving memory by slowing the forgetting. However, because we consider that these values can change continuously in different agent execution contexts and are controlled by other cognitive functions such as a top-down affect regulation system and an attentional system; establishing the values of alpha and beta is beyond the scope of this article.

(16) $$\begin{array}{c} w\left(t\right)=\alpha \cdot w_{1}+\beta \cdot w_{2}, \end{array}$$

where 0.0 < *α*, *β* < 1.0.

Finally, the general activation *a*_*f*_(*t*) represents the average between the previous general activation *a*_*f*_(*t* − 1) and the current activation *a*_*p*_(*t*), which is calculated using the sigmoid function, due to its resemblance to neuron spikes, over *w*(*t*).

(17) $$\begin{array}{c} a_{\left\{p\right\}\left(t\right)}=\frac{1}{1+e^{\left\{-w\left(t\right)\right\}}},\\ a_{f}\left(t\right)=\frac{a_{p}\left(t\right)+a_{f}\left(t-1\right)}{2}. \end{array}$$

The decaying function *d*_*a*_ modifies the base level of the storage components and decays over time. It is given by a Gaussian function that depends on the current activation of *a*_*p*_, and the parameter *γ* that controls the decaying speed.

(18) $$\begin{array}{c} d_{a}\left(t\right)=a_{p}\cdot e^{-\left(t^{2}/\gamma \right)}, \end{array}$$

where *γ* ∈ *N*_0_.

Additionally, the stored affective values for each object or scene can be updated. Thus, the new values for each affective parameter as_*ρ*_ ∈ Pa are the average affective values between the stored ones and those currently perceived.

(19) $$\begin{array}{c} {\text{as}}_{\rho }\left(t\right)=\frac{{\text{as}}_{\rho }\left(t-1\right)+{\text{as}}_{\rho }\left(t\right)}{2}. \end{array}$$

## 5. General Implementation of Each Module

The proposed architecture was developed using technologies such as Unity 3D, Image AI in Python, and a Java Framework for the development of cognitive architectures [143, 144]. Jaime et al. [144] and Cervantes [143] framework allowed us a straightforward, distributed, and concurrent implementation and was compatible with our approach of using an abstraction of the bran. This framework also performs the encoding and decoding of the spikes, which are data structures used to resemble the brain's action potential and are transferred among the components (see Table 1). Unity was used for the construction of the virtual environment, and Image AI for supporting object-recognition processes. The pain and pleasure values used to calculate the affective value depended on the labels of the dataset used by the Image AI object recognition method. Thus, the values corresponding to pain and pleasure were generated randomly, and both were assigned to each label.

The system considered a virtual agent placed inside the virtual world created in Unity 3D. The data captured from the agent's vision system were sent to the Python subsystem and processed with OpenCV [145] and ImageAI [141, 146] to help in visual processing. The extracted visual features (object class, location, and image) were sent to the subsystem built with the Java Framework. The sensed values from the body component were transferred directly to the Java framework.

**Figure 5:**
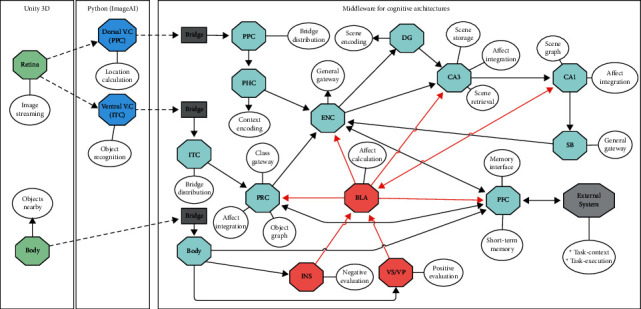
The implementation included three subsystems: a virtual environment (Unity 3D), an image processing subsystem (Python), and the core of the memory processes (implemented using the middleware). The Unity and Python modules communicated directly through image streaming, but the interaction between middleware and Python required a bridge that kept the data encoded. The octagons represent the model's components, and the ovals denote the processes carried out by those components. The external system was an independent type of component whose implementation varied with the case study.

To keep the distributed and modular approach, the Unity and Python subsystems were built following the same design proposed by Jaime et al. [144].  Figure 5 shows the interaction of these subsystems in our proposal.

### 5.1. Case Study

The case study presented in this paper consisted of three experiments designed to evaluate the proposed emotional memory system's capabilities. The first experiment focused on demonstrating the acquisition and retrieval of emotional associations. The second case showed the use of the acquired knowledge to solve a planning task. Finally, the third case showed how emotional data can improve learning in declarative knowledge. In all three experiments, the default values for alpha and beta were assigned to 0.25 and 0.75, respectively. It means that affect will improve memory, but retention will still depend mostly on experience by repetition.

#### 5.1.1. Experiment 1: Capabilities Test

The first case study showed the capabilities of the emotional memory system. It included two stages. During the first stage, a virtual agent had to wander around a scenario to acquire knowledge about the environment. The second stage consisted of evaluating these data and showing whether the agent was capable of learning and making affective associations.

#### 5.1.2. Learning

**Figure 6:**
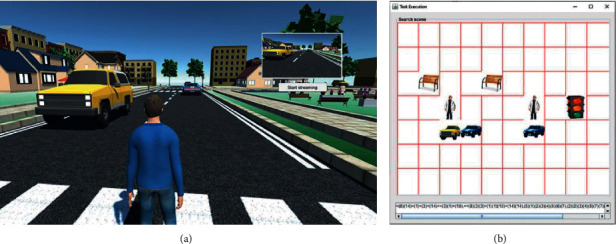
(a) The virtual world developed in Unity 3D. In this world, the agent wandered freely. The environment consisted of indoor and outdoor locations with different classes of objects. All the scenes seen by the agent are captured and encoded by the memory system. (b) Visual representation of the memory encoding of scenes in an m×n matrix. This matrix is used to generate pictorial queries to search for similar scenes and their affective values based on the positions of the objects.

In this stage, the virtual agent was immersed in an unknown 3D world. The virtual world consisted of multiple rooms with different objects that the agent needed to learn. Each object had a pain and pleasure value generated randomly before the execution. The agent was positioned in a random place and started to wander freely during a specific time. By the end, the agent learnt the rooms, objects, and the affective value associated with each one.  Figure 6(a) shows the virtual world.

#### 5.1.3. Retrieval

Once the agent had learned the scenario, a series of questions regarding the environment helped evaluate its knowledge. The questions were presented as a pictorial query (see Figure 6(b)) that would retrieve the affective value associated with the given scene.

#### 5.1.4. Experiment 2: Planning Task

The second experiment showed one of the possible applications of the proposed system. The main goal was to explicate how the agent used the acquired knowledge to perform a planning task. Like the previous case, it consisted of two stages. During the first one, the agent wandered and learnt about the environment, while in the second, the system asked it to go to a particular location. Thus, it had to plan how to get to the specified place.

#### 5.1.5. Learning

**Figure 7:**
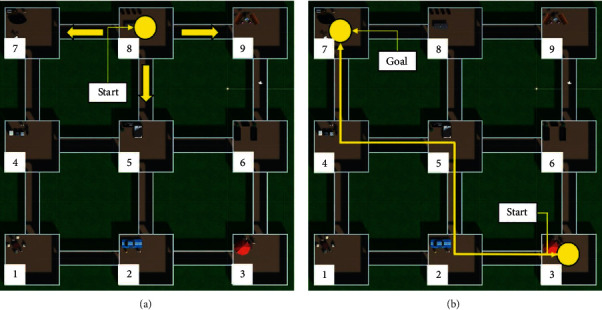
(a) Top-view of the virtual environment that the agent learned while wandering around the rooms. (b) The agent had to create a plan to move through the rooms to reach the goal.

This stage was like the one in the first experiment. Therefore, the agent had to wander in the virtual world to learn its content and make affective associations. In this case, the world consisted of 9 rooms with different objects. Each object had a pain and pleasure value generated randomly before the execution. The agent was positioned in a random room and started to wander freely for a specific time (see Figure 7(a)).

#### 5.1.6. Planning

In this planning stage, the agent was positioned in a room and instructed to go to a specific scene in the world. Therefore, it had to plan the route it was going to take to reach its goal. The planned route could consider or ignore the affective value of each room (see Figure 7(b)).

#### 5.1.7. Experiment 3: Reinforcement Task

**Figure 8:**
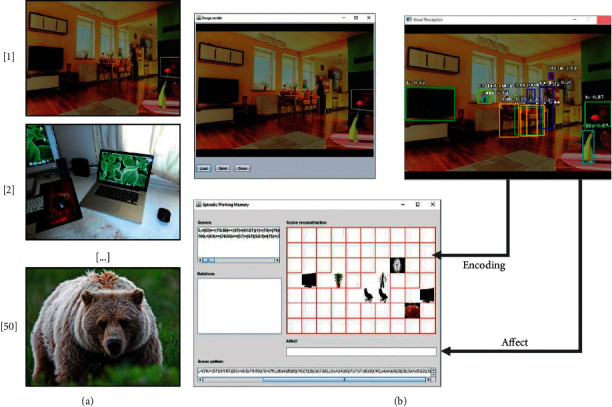
Representation of experiment. (a) Fifty images were presented to the system, and each one was considered as a single class. The pain and pleasure values for the classes were generated randomly before the presentation. (b) The images' affective value was calculated, and they were stored for future retrieval.

Finally, the third experiment demonstrated the improvements in memory behaviour caused by affection. The experiment included two stages. In the first stage, a series of neutral images was shown to the virtual agent; then, it had to remember some of the pictures. The second stage was identical to the first one, except that the images had been assigned affective values that could influence the retrieval (see Figure 8).

#### 5.1.8. Learning and Evaluation without Emotions

In this stage, a sequence of images was presented to the agent. After a specific amount of time, some queries were presented to the agent to evaluate whether it could remember the pictures.

#### 5.1.9. Learning and Evaluation with Emotions

This stage was identical to the previous case; a sequence of images was presented to the agent. However, this case differed in that each image was associated with an affective value that could influence memory consolidation. After a specific amount of time had passed, some queries were sent to the agent to evaluate whether it could remember the pictures.

## 6. Results

The results presented below focus on showing the feasibility of our system to perform memory and affective functions and to perform a simple cognitive task using affective knowledge.

### 6.1. Experiment 1

During the first stage, the agent was wandering in the virtual world for 30 minutes. The pleasure and pain values for each class (80 taken from the coco dataset [147]) were assigned randomly before the execution. These values were established in the range of (0.0–0.5). However, the pleasure values for the classes in the interval 15–24 (animals) were given values between 0.6 and 1.0. And, the pain values for the classes between 46 and 56 (food) were set with values between 0.6 and 1.0. This difference between values was carried out to show more clearly how the emotional system works.

**Figure 9:**
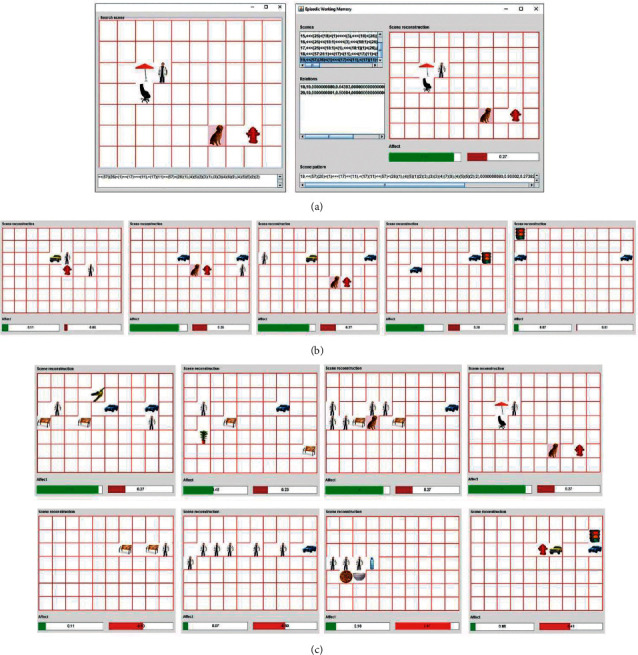
Results obtained after the execution of experiment 1. (a) The pictorial query (left) triggers the retrieval of the most similar scene that the agent saw and its affective values. (b) Influence of a highly affective object over subsequent memories. The presence of the dog increases positive affect (green bar) and decreases when the dog disappears. (c) Multiple memory traces and their affective associations. Some objects increase the positive affective value, while others, the negative one.

Figure 9 presents the results obtained from the queries performed after the execution of the first stage. Part (a) shows an example of how the queries were performed to trigger the retrieval of a scene and its affective values. In this case, the scene had (0.90, 0.27) of affection. Part (b) presents a set of scenes that were stored consecutively. The positive affective values assigned were 0.11, 0.85, 0.89, 0.66, and 0.07, respectively. Here, we can observe that the values started lower before the appearance of the dog, then there was a significant increase, and when it disappeared, the values decayed again. This increment means that the presence of a highly emotional object can influence subsequent memories. In part (c), the retrieved scenes had the pairs of values (0.94, 0.27), (0.46, 0.23), (0.90, 0.27), (0.90, 0.27), (0.11, 0.53), (0.07, 0.50), (0.16, 0.52), and (0.08, 0.49), respectively.

Based on these values and the scene content, we can see that the system correctly assigned the affective values. It means it followed the bias established for food and animals. The scenes that contained animals (dog and bird) or food (pizza and a bowl) had higher affective values than those that only had neutral objects.

### 6.2. Experiment 2

**Figure 10:**
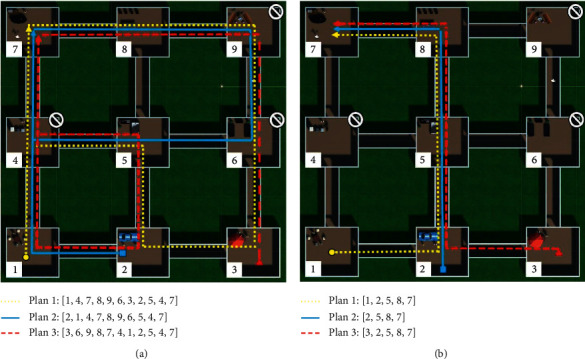
Results obtained after the execution of experiment 2. (a) Planned routes ignoring negative affective values. (b) Planned routes considering negative affective values.

During the first stage, the agent learned the room for 30 minutes. Each class's pleasure and pain values (80 taken from the coco dataset) were assigned randomly before the execution in the range (0.0–0.5). However, the object classes' pain values inside the rooms 4, 6, and 9 were between 0.8 and 1.0, and the pleasure values were in the interval 0.0–0.3. The first learning column in Table 2 shows the average affective values for each room after the execution of the first stage. The planning stage started with the agent instructed to go to room seven, starting from rooms 1, 2, and 3 in three different executions and ignoring all the rooms' affective values. These executions were then repeated but considering the negative values below a threshold of 0.75.  Figure 10 presents the results of the planning stage. In part (a), we appreciate that the paths indiscriminately passed through the negative rooms, while in part (b), the paths avoided passing through those rooms.

**Figure 11:**
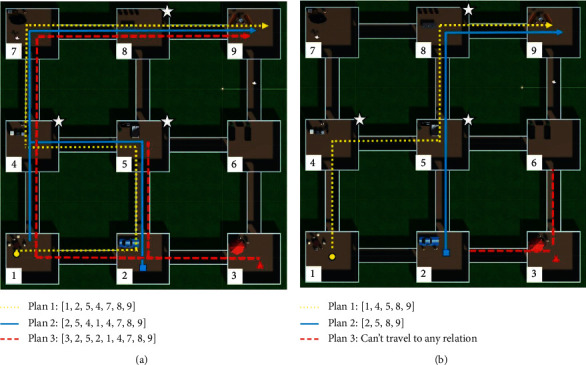
Results obtained after the execution of experiment 2. (a) Planned routes ignoring positive affective values. (b) Planned routes considering positive affective values.

The complete experiment was repeated, starting from a second learning stage. In this stage, the pleasure and pain values remained in the range (0.0–0.5). However, the object classes' pleasure values inside the rooms 4, 5, and 8 were between 0.8 and 1.0, and the pain values were in the interval (0.0–0.3). The second learning column in Table 2 shows the average affective values for each room after the execution of the first stage's repetition. The planning stage started again with the agent instructed to go to room 9, starting from rooms 1, 2, and 3 in three different executions and ignoring all the rooms' affective values. Then, these executions were repeated, but considering the positive values above a threshold of 0.75.  Figure 11 presents the results of the planning stage. In part (a), we observe that the paths indiscriminately passed through the rooms. In contrast, in part (b), the paths avoided passing through those rooms. Nevertheless, when the agent started in room number three, he could not find a way because the connected rooms had a positive value below the threshold.

### 6.3. Experiment 3

The first stage took 50 images from the coco dataset [147]. We divided this stage into two executions. For the first execution, the images had a pain and pleasure value of zero, and for the second one, values were between 0.0 and 1.0. In both runs, 50 images were presented to the system one by one every 10 seconds. After the completion of each execution, the system stopped for 5 hours before the evaluation.

**Figure 12:**
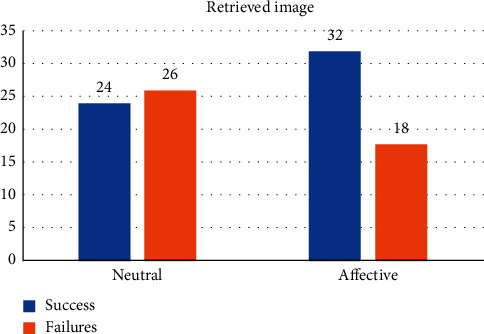
Results obtained after the execution of experiment 3. (a) Mean values obtained for neutral images. (b) Mean values obtained for affective images.

During the evaluation stage, the system tried to retrieve the 50 images stored with neutral values during the first execution and the 50 images with affective values from the second execution.  Figure 12 shows the results obtained after the evaluation of 10 pairs of executions. It can be observed that the average of successful retrievals (24) against forgotten images (26) is lower when the image has a neutral value of affection. However, when the image has an affective value associated with it, either positive or negative, it has a higher score of successful retrievals (32) than failures (18). These results mean that our system can, in a simple way, simulate the retrieval improvements caused by emotions.

## 7. Discussion

Let us start by discussing the results obtained by the implementation of the proposed model. With experiment 1, we demonstrate the emotional memory system's capabilities for storing and retrieving information during the virtual agent's interaction with the stimuli perceived in the environment. In this experiment, we observed how the system correctly assigned affective evaluations to the scenes that it was learning based on the biases suggested. Also, when a scene had a very high rating, it affected subsequent scenes just as it does. However, the decay values that produced this behaviour were arbitrarily selected, so it is necessary to carry out more experiments that will allow us to adjust them and have results that are comparable with those of humans. Also, from neuroscientific evidence, we believe that the definition of these values requires interaction with other cognitive systems such as attention, top-down emotions, planning, and decision-making. Moreover, the interaction with each of those systems is an ongoing work that is beyond of the presented work. Experiment 2 sought to demonstrate the relevance of considering the integration of emotions with memory in a planning and decision-making process. In this case, we were able to show how affection can bias the virtual creature to avoid or give preference to specific situations. In most situations, this bias caused an improvement in both processes and made it impossible in one (because the connected rooms were not positive enough to be chosen). As in the previous case, an adjustment is required in the decay parameters. Given the time and size of the environment, it is likely that more realistic values would cause all memory traces to be affected by a room's affective value, and there would be no clear difference between them. Finally, experiment 3 aimed to validate that affective evaluations' intensity improves the learning process by reinforcing knowledge in memory. The results obtained show that those memory traces with higher valuations are more difficult to forget than those with a neutral value. Although these results keep correlation to a certain level with experiments in humans, more experiments are also necessary to allow us to adjust certain parameters such as the influence weight of emotions concerning memory, the forgetting scale of the memory system, and the threshold of the recoverability level. We believe that adjusting the parameters of the cases will allow the system to generate behaviours directly comparable with those of a human.

Overall, the results obtained from the three experiments helped us to demonstrate our system's capabilities and feasibility and to conclude that the proposed model is functional for encoding memory traces and their affective values from the stimuli perceived in a virtual environment or simulations. These values help the virtual creature to generate autonomous behaviour, improve its decision-making process, and reinforce its learning. Also, from the results of experiment 3, we can observe that if we adjust the correct parameters in the system, we can reach realistic results such as those presented by Marchewka et al. [148].

Nevertheless, it should be noted that some functionalities could be added or improved to make the system more complete and closer to biological evidence. Even though the general model presented in Section 4 shows all the relations of the affect, the developed model still lacks the top-down regulation of emotions that would allow for conscious control and modification of affective values. Currently, our model uses bottom-up information coming from the environment to create affective associations, which means that similar situations can have similar affective values. However, if we include emotional regulation, our model can use other cognitive functions such as planning, decision-making, attentional control, and a reward system to bias the emotions according to additional criteria. Also, additional work is required to cover the fast retrieval of memories with high affective values. Furthermore, this work focused on the association of episodic memories with emotions. Because episodic memories are a type of declarative or conscious memory, the behaviours generated by our system are also conscious. Therefore, extensive research is required to cover automatic behaviours such as the alarms proposed in [149].

Moreover, we indiscriminately use positive and negative values for memory improvement. Thus, the model can be extended to consider a different level of reinforcement depending on the type. Furthermore, the addition of brain structures to the model, such as stress-related areas and the hypothalamic-pituitary-adrenal axis (HPA), can endow the system with real properties like amnesia caused under stressful situations.

For its part, the proposed model based on biological evidence for the cognitive function of emotional memory proves to be consistent in storing and retrieving affective evaluations. In the same way, it is functional in generating plans based on affective information. Although affective evaluations are the first step for the storage of emotions, there are still other functionalities for the general storage of emotional states. In particular, the storage of emotional states such as joy, sadness, anger, and displeasure, among others, seems to follow the same storage circuit, leading to an overlap between brain structures. However, it seems that they differ in the point of generation of these emotions within the human brain. Such investigation and identification of brain structures involved in a specific emotional state are part of our team's current work. From the perspective of memory, we have observed that the proposed model of episodic memory with emotions is a good starting point for gradually generating a general architecture of declarative emotional memory. Many of the included areas can be extended in functionality to consider other types of memory, such as semantic and spatial memory. This last point is very important since it allows for the generation of a map of the environment that could lead to more complex behaviours when combined with the knowledge acquired in our proposal. As with the case of emotions, these extensions will be part of future research.

The proposed system makes evident the need to implement the emotional memory process in cognitive architectures. This process helps to skew decisions and support the survival of cyber entities. Even though this process was not observed in the identified cognitive architectures (see Section 2), the interaction between memory systems and the emotional system is observed in some of them [8, 9, 11, 12]. Reinforcing this cognitive process of emotional memory in other architectures could improve the behavioural responses exhibited by cybernetic entities, getting a little closer to the behaviour of their biological counterparts, human beings.

Finally, we have verified that the combination of memory and emotion functions will have an extensive research line, as we have demonstrated the great importance of these mixed processes by implementing a system aimed at storing affective evaluations of perceived stimuli. The future research panorama is much broader, ranging from how specific emotional states are stored (such as sadness, joy, and anger) to how cybernetic entities behave through emotional regulation, i.e., when they face known situations, how they control their behaviours and their emotional responses to the environment. Given the evidence collected in this work, we can say that emotional memory is a complex matrix process that involves different types of emotional evaluations with different levels of cognition requiring further research.

## 8. Conclusions

Episodic memory and emotions are essential functions for human beings because they allow us to acquire knowledge from the environment related to our daily events or situations. This knowledge is essential for computing our behaviour. Furthermore, these systems help us identify dangers and rewards in the environment, thereby biasing decisions. For these reasons, both are highly desired functions for virtual agents if they need to show human-like behaviour. Researchers have been working to design cognitive architectures that include both processes and endow agents with human-like capabilities.

This study presents a cognitive architecture proposal that considers and integrates episodic memory with the affective part of the emotions. The architecture design is grounded in psychological and neuroscientific evidence, which provides information on the components and the processes carried out by memory and affection. Unlike other proposals, this study uses a modular and distributed approach providing it scalability, resilience, and modularity characteristics. This last characteristic allows the proposed components to be replaced or extended in order to include new evidence. Although this proposal was designed to be part of a broader cognitive architecture, it can also work independently or be used in other projects. Finally, we are aware that our system is in the early stages of development and requires more experiments. We consider that future versions can work as a testbed for multiple investigations in cognitive architectures and intelligent agents.

## Figures and Tables

**Table 1:** The spike structures built from the data described in the process formalization of Section 4.

Spike name	Data structures	Description
S1	(*I*, *t*)	Image captured from the environment
S2	(*C*, *t*)	List of objects' positions
S3	(*O*, *t*)	List of objects' classes
S4	(Scene_*p*_, *t*)	Context pattern
S5	(*O*, *t*)	List of objects' classes
S6	(Scene_*a*_, *t*)	Scene pattern
S7	(*G*_*s*_, *t*)	Scene relations
S8	(*O*, *t*)	List of objects' classes
S9	(ES_pain_, *t*)	Negative evaluation
S10	(ES_pleasure_, *t*)	Positive evaluation
S11	(as_pain_, as_pleasure_, *t*)	Affective values
S12	(*G*_*o*_, *t*)	Objects' relations
S13	(2DString_*T*_, *t*)	Scene pattern
S14	(Id, *t*)	Scene ID, where Id ∈ *ℕ*
S15	(Id, *t*)	Object ID, where Id ∈ *ℕ*

**Table 2:** The average affective values calculated for each room. The first learning column shows the values calculated with the data skewed to pain, and the second learning column shows the values skewed to pleasure. Numbers in bold represent the highest values.

First learning			Second learning	
Room	Positive	Negative	Positive	Negative
1	0.35	0.71	0.59	0.17
2	0.44	0.44	0.35	0.21
3	0.33	0.60	0.14	0.14
4	0.32	**0.87** ^*∗*^	**0.76** ^*∗*^	0.15
5	0.37	0.35	**0.76** ^*∗*^	0.16
6	0.43	**0.79** ^*∗*^	0.32	0.19
7	0.39	0.53	0.52	0.16
8	0.44	0.51	**0.80** ^*∗*^	0.15
9	0.33	**0.83** ^*∗*^	0.47	0.14

## Data Availability

No data were used to support this study.
